# A scoping review and behavioural analysis of factors underlying overuse of antimicrobials

**DOI:** 10.1093/jacamr/dlad043

**Published:** 2023-05-08

**Authors:** Olga Poluektova, Deirdre A Robertson, Aisling Rafferty, Robert Cunney, Peter D Lunn

**Affiliations:** Behavioural Research Unit, The Economic and Social Research Institute, Whitaker Square, Sir John Rogerson’s Quay, Ireland; Trinity College Dublin, School of Social Science and Philosophy, Department of Sociology (Visiting Research Fellow), Dublin, Ireland; Behavioural Research Unit, The Economic and Social Research Institute, Whitaker Square, Sir John Rogerson’s Quay, Ireland; Trinity College Dublin, School of Psychology, Dublin, Ireland; Children’s Health Ireland, Department of Pharmacy, Dublin, Ireland; University of Birmingham, School of Pharmacy, Institute of Clinical Sciences, Birmingham, UK; Children’s Health Ireland, Irish Meningitis and Sepsis Reference Laboratory, Dublin, Ireland; Royal College of Surgeons in Ireland, Department of Microbiology, Dublin, Ireland; Children’s Health Ireland, Department of Microbiology, Dublin, Ireland; Behavioural Research Unit, The Economic and Social Research Institute, Whitaker Square, Sir John Rogerson’s Quay, Ireland; Trinity College Dublin, Department of Economics, Dublin, Ireland

## Abstract

**Background:**

Overuse of antimicrobials is a challenging global issue that contributes to antimicrobial resistance. Despite widespread awareness of the problem among members of the medical community and various attempts to improve prescription practices, existing antimicrobial stewardship programmes are not always effective. In our view, this may reflect limited understanding of factors that influence prescription of antimicrobials as empirical therapy, implying a need to address the psychological mechanisms behind some of the specific behaviours involved.

**Objectives:**

To identify factors that influence the antimicrobials prescription as empirical therapy, and to relate these factors to findings from behavioural science.

**Methods:**

We conducted a scoping review of the literature on the factors underlying antimicrobial prescription decisions, following the protocol designed using PRISMA guidelines.

**Results and conclusions:**

From a final sample of 90 sources, we identified ten factors important in antimicrobial prescription decisions. In the second stage of our analysis, we grouped them into five final categories: (1) nature of the decision, (2) social influences, (3) individual differences, (4) characteristics of the patient, (5) context. We analyse these categories using a behavioural science perspective.

## Introduction

Overuse of antimicrobials is an increasingly challenging global issue.^1^ We use the term ‘overuse’ to refer to the use of antibiotics not supported by evidence or existing guidelines, which contributes to the development of drug-resistant pathogens. Taking antimicrobials when they are not absolutely necessary and not following the prescription regimen contributes to antimicrobial resistance, which leads to higher medical costs, prolonged hospital stays and increased mortality.^2^ Antimicrobial resistance is now a leading cause of death worldwide.^3^ One way that we might slow down bacterial resistance is changing attitudes and behaviour related to the use of antimicrobials.

To address overuse of antimicrobials, antimicrobial stewardship programmes have been introduced.^4–8^ While such programmes help to improve prescription practices and lead to more prudent use of antimicrobials, as well as higher general awareness of the problem among medical students^9^ and practising physicians,^10,11^ the problem of overuse persists. For instance, across Europe, antimicrobial prescriptions were inappropriate or inconclusive in one-third of cases of febrile children admitted to emergency departments.^12^ Meanwhile, in the USA, while physicians regard antimicrobial overuse as a national health crisis, they severely underestimate the prevalence of the problem in their own facility.^13^

In order to improve existing approaches and devise new solutions for tackling the problem of overuse, it is important to understand what causes it. A large body of literature provides evidence that medical decisions are susceptible to biases and errors in diagnosis and treatment.^14–16^ Physicians are vulnerable to many classic biases commonly discussed in behavioural economics and social psychological literature, including the representativeness and availability heuristics,^17^ confirmation bias,^18–20^ risk and uncertainty avoidance.^21,22^

These phenomena likely apply in the context of antimicrobial prescribing as well. Indeed, antimicrobial prescription decisions have been previously linked to cognitive and motivational biases.^23,24^ There is, however, scope for a more comprehensive review of existing literature and an in-depth analysis of the possible mechanisms involved.

Although studies have asked which factors might be important in prescription decisions, little work has sought to systematize the results and offer a more complete behavioural analysis. For example, Teixeira Rodrigues and colleagues,^25^ based on a systematic review of qualitative research on the topic, proposed a categorization of factors important in prescription behaviours. This framework gives a good initial overview of *what* might be at play in antimicrobial prescription decisions but does not take account of quantitative evidence and stops short of identifying mechanisms that would explain *how* and *why* certain factors translate in prescription decisions. Our belief is that the answers to these questions are crucial for a more in-depth understanding of antimicrobial overuse, giving us a better chance to improve prescription practices and slow down antimicrobial resistance.

In response to this gap in the literature, this article seeks to identify factors central to antimicrobial prescription decisions and to analyse them from a behavioural science perspective. To achieve this aim, we conducted a scoping review of the literature on factors influencing decisions to prescribe antimicrobials as empirical therapy. That is, we focused on the influences on prescription decisions made under uncertainty, where prescribers lack precise information, such as the result of a microbial investigation that indicates a specific bacterium or fungus causing the infection, and where no clear guideline for how this infection should be treated exists. Such influences include both clinical factors (e.g. severity and duration of symptoms) and non-clinical factors (characteristics of the prescriber or the environment in which the prescription takes place). We did not include studies that focused solely on the results of microbial investigation as a factor in prescription decisions. Based on the results of this review, we developed a framework that includes five categories of influences on prescription decisions: (i) nature of the decision; (ii) social influences; (iii) individual differences; (iv) characteristics of the patient; and (v) context. We analyse each category from a behavioural science perspective. It is our hope that the results of this more in-depth analysis can inform the design of new effective solutions for improving prescription practices.

## Methods

We developed our review protocol following PRISMA guidelines for scoping reviews.^26^ One researcher (the first author) drafted the protocol that was further discussed and revised by the whole team of authors. The full document with the research protocol, as well as the final list of sources included in the review, can be accessed at https://osf.io/eg2ck/files/osfstorage/63ebcb66b3fed60527e34290.

When developing our search strategy, inclusion and exclusion criteria, we aimed to achieve as comprehensive an overview of the factors contributing to antimicrobial prescriptions as possible. Thus, we did not limit our search to any particular geographical location, time period, healthcare setting, medical profession of the prescriber or symptomatology. We assumed that antimicrobial prescription decisions, although affected by contextual factors that may vary between healthcare settings and geographical locations, have a substantial degree of universality across different settings. We list the inclusion and exclusion criteria below.

### Inclusion criteria

We considered study design, outcomes and the fact that the study was focused on the predictors of prescription decisions in determining our inclusion criteria. We included qualitative and quantitative studies. The outcome of interest was prescribing antimicrobials for empirical treatment, in the absence of an accurate microbiological diagnosis. As predictors, we sought articles that focused on factors other than microbial investigation, that influence prescription decisions. As we aimed for as comprehensive an overview of such factors as possible, we chose to follow an inductive approach and did not list specific characteristics as keywords, so as not to limit our search for relevant factors. We included all articles that discussed factors that influence decisions to prescribe antimicrobials.

### Exclusion criteria

Not all articles found were selected for the analysis. First, we excluded articles if the focus was not human medicine (e.g. veterinary or farming). Second, articles were not included if the main focus was on patients’ attitudes and behaviours, and they did not incorporate the prescriber’s perspective. Third, articles were left out if they reported research conducted on specific patient populations that are at a higher risk of infection—perioperative, oncology and immunocompromised patients—as these groups are often recommended to be prescribed antibiotics by default. Fourth, we did not include articles that reported studies focused solely on the results of microbial investigation as a predictor of prescription behaviours. Finally, we did not include systematic reviews and randomized control trials (RCTs) that tested solutions aimed at improving prescription behaviours. Systematic reviews summarize individual studies, which we incorporate in the review and analysis anyway. As for RCTs, although interventions evaluated as part of RCTs are often designed to influence factors that relate to prescription behaviours, they typically cover multiple such factors at once, making it impossible to differentiate between the effects of different factors or measure their individual contribution to decision-making and behaviour.

### The search

The search was conducted using MEDLINE (utilizing PubMed and PubMed Central search engines), ScienceDirect and APA PsycInfo. MEDLINE was chosen as one of the most comprehensive and frequently used databases for research on medical topics. ScienceDirect was chosen as one of the world’s leading sources for scientific, technical and medical research. APA PsycInfo was selected as the most trusted database for psychological research, as we were interested in human decision-making and behaviours and factors and mechanisms explaining them. The search and selection of the sources for the review were conducted by one reviewer (the first author).

We used the following script: *(antibiotic prescribing [Title] OR antimicrobial prescribing [Title] OR decision to prescribe antibiotics [Title] OR decision to prescribe antimicrobials [Title]) AND (factors[Title/Abstract] OR determinants[Title/Abstract] OR drivers[Title/Abstract] OR correlates[Title/Abstract] OR predictors [Title/Abstract] OR antecedents [Title/Abstract])*.

### Analytical approach

The selection of the final sample of sources was conducted in two steps. First, we screened the titles and abstracts of the sources and eliminated a large proportion of them based on our exclusion criteria. Second, we eliminated an additional portion of sources after reading the full texts of the articles. The screening was done by the first author.

Our analytical approach was inductive; we planned to derive the framework of factors influencing prescription decisions from the data. This meant that we did not have any preconceived notions of what the codes—the main themes—used to document and classify the sources should be. To develop the codes, we selected 10 (approximately 10% of the sample) articles that would further allow us to document and classify the factors influencing antimicrobial prescriptions on the full sample of sources. To ensure that the codes were exhaustive, we selected articles reporting on studies with different methodologies [two experimental, two qualitative and six correlational, set in different settings (inpatient, emergency department, outpatient, long-term care facility), different patient populations (adults, children) and different geographical locations (Europe, USA, Asia). The distribution reflects the distribution of the articles reporting on experimental, qualitative and correlational studies in the full sample of final sources. Based on the analysis of the selected 10 sources, we identified 10 factors important to antimicrobial prescription decisions. The remainder of the material was coded using these factors. The coding was performed by one researcher (the first author).

In the final stage of the analysis, after a thorough discussion between three researchers (the first, second and last authors), followed by oversight and agreement by the third and fourth researchers, it was decided to group the 10 factors into five final categories based on the similarities between the factors and underlying psychological themes.

## Results

### Source characteristics

**Figure 1.**
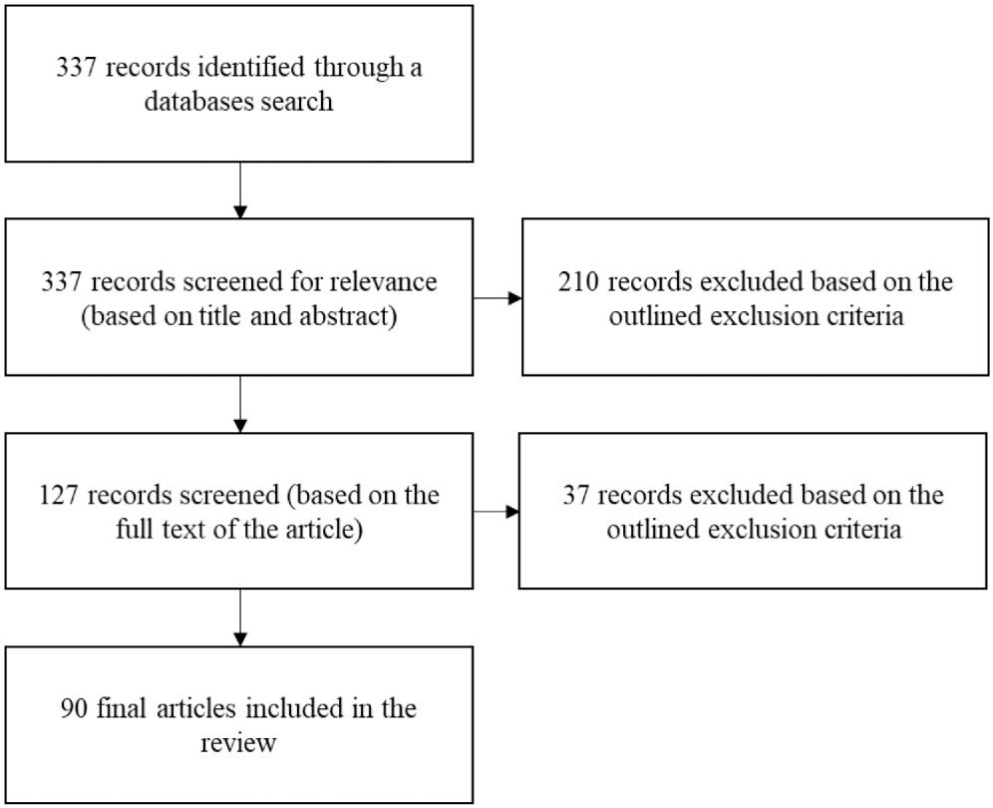
Number of identified and excluded sources.

Our search identified 337 sources; 285 sources were found using PubMed, one using PubMed Central, and 51 using ScienceDirect. The final sample of sources included 90 articles. Figure 1 maps out the number of records identified and excluded at different stages of the search and analysis process.

The large majority—68 articles (75% of the total pool)—reported on quantitative studies that used correlational designs. Research carried out in North America featured in 36 articles (40%), followed by the studies conducted in European and Asian countries. Most sources (70 articles, which is more than 75%) presented research conducted in outpatient primary care settings. Half of all studies featured prescriptions for respiratory infections, although in a large proportion of the articles no focus on specific symptomatology or condition was made. While 16 sources reported on research featuring prescriptions for paediatric patients, most were not limited to a specific patient group. Table 1 presents a summary, with the main characteristics of the sources identified.

**Table 1.** Summary of the sources included in the review

	Quantitative	Qualitative
Methodology		
* *Experiment	2	NA
* *Survey/correlational	68	NA
* *Interview	NA	13
* *Focus group	NA	10
* *Total	70	21
Setting		
*** *** Outpatient**—**primary care	56	12
*** *** Outpatient**—**specialty care	4	
*** *** Outpatient**—**emergency care	15	3
*** *** Inpatient care	4	5
*** *** Long-term care facilities	1	2
Condition		
*** *** Respiratory infections	36	10
*** *** Common cold	4	
*** *** Urinary tract infections	2	
*** *** Acute otitis media	2	
*** *** Not specific to any condition	31	11
Patient population		
*** *** Children	13	4
*** *** Adults	4	
*** *** Elderly	2	2
*** *** Non-specific	51	15
Region		
*** *** EU	13	4
*** *** North America	35	6
*** *** UK	4	4
*** *** Asia	12	5
*** *** Australia	2	2
*** *** Middle East	2	
*** *** Africa	1	

Some studies included more than one research design, were conducted in more than one setting and focused on more than one condition. NA, not applicable.

### Factors in antimicrobial prescription decisions

**Figure 2.**
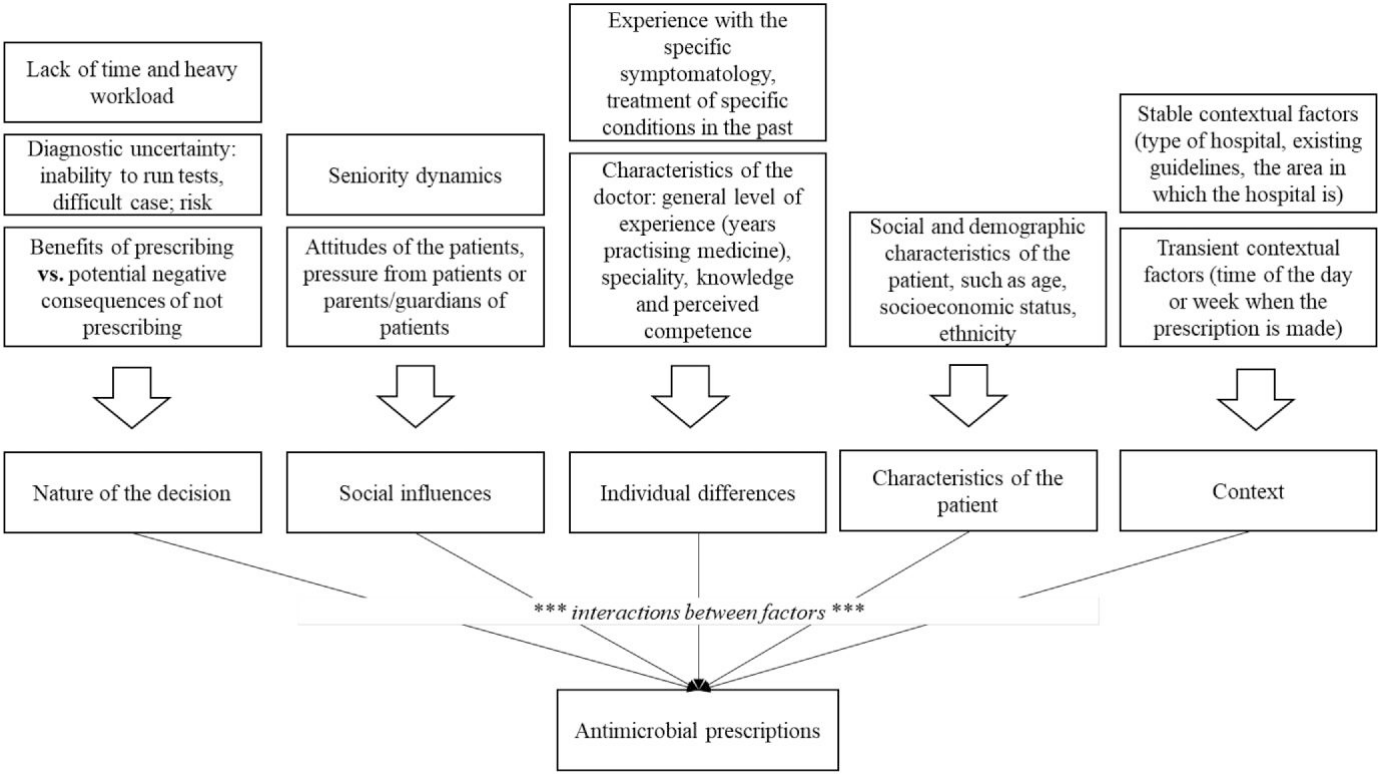
Five categories of influences on antimicrobial prescription decisions.

Based on the analysis of the selected articles, we identified 10 factors important in antimicrobial prescription decisions (Table 2). These factors informed our framework of influences on prescription decisions. The framework consists of five categories: (i) nature of the decision; (ii) social influences; (iii) individual differences; (iv) characteristics of the patient; and (v) context. Figure 2 shows how the 10 factors form the five categories of influences. In the next section, we analyse these categories from a behavioural science perspective.

**Table 2.** Factors playing a role in prescription decisions

Factor	Number of sources featuring the factor	Sources	Final category
Characteristics of the doctor: general level of experience (years practising medicine), speciality, knowledge and perceived competence.	29	^27–55^	Individual differences
Social and demographic characteristics of the patient, such as age, socioeconomic status, ethnicity.	27	^32,37,42,44,47,49,54–76^	Characteristics of the patient
Attitudes of the patients, pressure from patients or parents/guardians of patients.	26	^28,35–37,46,48,51,55,58,59,77–94^	Social influences
Stable contextual factors (type of hospital, existing guidelines, the area in which the hospital is).	24	^34,45,47,48,53–55,63,69,70,72,73,77,79–81,91,95–102^	Context
Diagnostic uncertainty: inability to run tests, difficult case; risk.	21	^28,31–33,55,58,59,72,78,80,81,83,91,94,103–107^	Nature of the decision
Lack of time and heavy workload.	14	^27–30,56,57,77–79,81,95,108–111^	Nature of the decision
Benefits of prescribing versus potential negative consequences of not prescribing.	5	^37,83,103,107,112^	Nature of the decision
Experience with the specific symptomatology, treatment of specific conditions in the past.	4	^50,71,113,114^	Individual differences
Transient contextual factors (time of the day or week when the prescription is made).	3	^50,115,116^	Context
Seniority dynamics.	2	^34,60^	Social influences

## Behavioural analysis of the factors underlying prescription decisions

### Nature of the decision

#### Lack of time and heavy workload

In clinical settings, decisions often need to be made under time pressure. Prescribers deal with many patients at the same time and are under pressure to come up with an accurate diagnosis and appropriate treatment plan quickly. Many articles included in our review feature time scarcity as an important factor in prescription decisions.^27–30,56,57,77–79,95,108–111^

Lack of time might contribute to a scarcity mindset,^117–119^ in which reduced mental bandwidth makes an individual more invested in immediate outcomes, without enough cognitive resources for thinking about other, less pressing, problems that might arise in the future. In the context of antimicrobial prescriptions, the immediate outcome is making the patient feel better as quickly as possible, while antimicrobial resistance is a more distant problem in the future that is not at the centre of the prescriber’s attention. In addition, scarcity and depleted cognitive resources likely make one more susceptible to other decision-making biases.^120^

#### Uncertainty, risks and benefits of prescribing

Our analysis showed that risk^28,31,32,58,80,103^ and uncertainty^33,59,81,82,104–106^ are among the leading predictors of overuse. Prescribers deal with multiple risks as part of their job, which might place the need for security—one of the most basic psychological needs—high on the list of their priorities. Exposure to multiple potential threats is an unavoidable part of the job, and, if a mistake is made, the stakes can be high. In an attempt to insure against threats (e.g. the development of a serious infection, patients’ complaints etc.), prescribers might prescribe an antimicrobial, viewing it as a more secure option. In line with this, the literature that we analysed suggests that when prescribing antimicrobials, prescribers are driven by the motivation to minimize negative feelings, such as anxiety related to missing an infection,^104,108^ on the one hand, and maximize anticipated benefits of prescribing^83,103,105,108,112^ on the other.

Lack of control, uncertainty and other negative emotions experienced by prescribers can lead to commission bias.^121^ Inaction symbolizes lack of control and power over a situation; committing an action is a way to gain control and cope with powerlessness.

### Social influences

Our review showed that interaction and relationships with colleagues^34,60^ and expectations of patients^28,35–37,58,59,77,79–90^ are important contributors to prescription decisions, suggesting that social interactions are important aspects to consider when discussing the problem of antimicrobial overuse. Humans are social animals, and maintaining positive social relationships is vital to their wellbeing.^122^ Thus, they strive to form and maintain these relationships and to be accepted as members of social groups important to them. Motivation to maintain good relationships and be accepted by members of significant social groups is associated with higher conformity with the views and beliefs shared by the members of this group.^123^ Driven by this motivation, prescribers might not be willing to express disagreement with a senior colleague or deny prescriptions to patients requesting them.

### Individual differences

Individual differences are characteristics that vary from prescriber to prescriber, such as specialty and experience, age or confidence in their clinical skills. Our review showed that older prescribers,^27,29,30,38–40^ physicians^41^ compared with nurse practitioners and physician’s assistants, international graduates,^29,40,42,60^ urban-practising physicians,^111^ self-dispensing physicians or those with a pharmacist on site,^111^ prescribers indifferent to changes^43^ and, in the case of prescriptions to children, non-paediatricians^44^ are more likely to prescribe antimicrobials compared with comparator groups. Furthermore, good clinical knowledge and clinical competency lead to lower rates of antimicrobial prescribing.^37,83,100,118,119^ Internal medicine physicians are more likely to prescribe broad-spectrum antibiotics.^47^ Finally, prescribers with high levels of uncertainty avoidance prescribe antibiotics more often,^112^ while those who believe in shared decision-making^48^ and have high confidence in their ability to apply the prescription guidelines^28,33^ prescribe less.

Considering these factors is important, not only because they can directly influence decisions of prescribers, but also because they might moderate the strength of bias associated with universal motives that we discussed previously. For instance, dispositional uncertainty avoidance might make one more prone to experiencing negative emotions in ambiguous situations, while high self-efficacy and confidence in one’s clinical skills might help to resist social pressure.

### Characteristics of the patient

The review confirms that characteristics of patients matter when it comes to prescription decisions. Antimicrobials are more often prescribed to males,^49,61,62^ patients with a chronic complex condition^63^ and comorbidities,^59,64^ patients with a history of smoking,^65,66^ patients who report a longer duration of symptoms or worsening symptoms,^62^ and older patients,^60,67^ including in paediatric contexts.^44^ Additionally, there are substantial ethnic and racial differences among patients receiving antibiotic prescription, with non-whites^65,68,69^ and patients with indigenous backgrounds^60^ being prescribed antibiotics more frequently. Finally, socioeconomic background and the level of deprivation of the patient matter for prescriptions too—patients with lower socioeconomic status are more likely to receive a prescription.^70,71,108^

It is likely that these factors lead to higher prescriptions as prescribers associate them with an increased risk of more serious infection and/or complications. For example, prescribers may know that non-white ethnicity and economic disadvantage are associated with living in overcrowded accommodation that increases one’s chances to become infected. Such heuristics can be useful as they help to reduce the amount of thinking and the time doctors spend with patients; however, overreliance on them can lead to unnecessary prescriptions.

### Context

#### Stable contextual characteristics

The reviewed literature shows significant variation in antibiotic prescription rates by location, economic deprivation of the area and type of practice. Rural^72,73,112^ and more deprived^96,97,112^ areas have higher levels of prescriptions. In Italy, more antimicrobial prescriptions are made in the southern regions,^95,98^ which can be explained by economic deprivation of the area too. In the USA, more prescriptions are made in the South and Northeast,^47,69^ and in areas with a high density of providers and clinics, potentially due to competition.^99^ Additionally, single-handed^97^ and non-training^97,100^ practices, as well as practices with limited resources,^34,70,79^ have higher prescription rates. On the other hand, having consistent patterns of prescribing within the practice, supportive practice policies, and enough resources such as consultation time, contribute to more prudent antimicrobial use.^87^

#### Transient contextual characteristics

Some sources indicate that prescription decisions depend not only on *where* a prescriber works (i.e. in which country/hospital), but also *when* they make decisions. Timing is important to consider as prescribers’ levels of stress and busyness depend on the time of the day or week, with potential implications for decision-making. For example, prescriptions on Fridays are significantly more frequent compared with other working days.^115^ Time of the year is also important,^50,116^ as prescribers might experience heavier workload during times when viral infections peak, leading to time scarcity and higher cognitive load. As an illustration, Gana and colleagues discuss how medical decision-making might be susceptible to biases in the context of the current COVID-19 pandemic.^124^

## Discussion

Antimicrobial resistance is a serious problem, but existing solutions designed to tackle the overuse of antimicrobials—one of the main drivers of resistance—have limited impact. To understand how they can be improved, it is important to know why overuse occurs in the first place. We observed that existing analyses of the factors in antimicrobial prescription rarely focus on the behavioural mechanisms that might explain *how* and *why* certain factors translate in specific decisions and behaviours.

To address this gap, we conducted a scoping review of the research on factors of antimicrobial prescription decisions when such decisions are made in the absence of evidence of bacterial infections. Based on the results, we propose a framework that allows for a behavioural analysis of factors central to prescription decisions. The antecedents of antibiotic prescription decisions can be classified into five categories of influences: (i) nature of the decision; (ii) social influences; (iii) individual differences; (iv) characteristics of the patient; and (v) context.

Our review and analysis above confirm the complexity of decisions to prescribe antimicrobials as empirical therapy, highlighting the variety of factors that influence the decision-making of prescribers. When it comes to targeting these factors via antimicrobial stewardship programmes, we believe there is no one-size-fits-all solution. Antimicrobial stewardship programmes exist in many clinical practices but not all practices are equally effective and antimicrobial resistance continues to rise. According to current predictions, 10 million people will die in 2050 as a result of antimicrobial-resistant infections.^125^ Some successful stewardship interventions may tackle some of the factors we have described in this review. For example, one stewardship programme found beneficial results in one clinical team by leveraging teamwork and social influences.^126^ Finding ways to expand on and scale local interventions such as these may help to improve stewardship programmes worldwide. This may be helped by adopting a more tailored approach involving a variety of different solutions. This review aims to initiate that process by identifying the main factors that could be targeted to help tailor solutions. While the discussion of specific solutions is beyond the scope of this article, our review uses existing evidence to identify factors influencing prescribing behaviour that successful programmes may need to consider. In doing so, we highlight them as topics for future research and for the attention of policy and practice within this domain.

Our review has limitations. First, there is an element of subjectivity as the literature search and coding was done by one person, which is not typical of a systematic review. However, our approach still satisfied the requirements for a scoping review,^127^ and we believe this subjectivity is not critical as our goal was not to make definitive conclusions about the prevalence and relative importance of different factors underlying prescription decisions, but rather to provide a broad initial overview of all possible influences and explain how they actualize in prescription decisions. Second, we included qualitative studies that do not assess the statistical significance or size of effects, making it difficult to conclude whether a given factor is an important predictor of prescription decisions. However, in our case, all factors featuring in qualitative studies were also identified in articles presenting quantitative research. The advantage of incorporating qualitative studies in the review and further analysis is that they pay attention to mechanisms that might explain how certain factors actualize in specific behaviours. Third, the majority of our sources focused on antimicrobial prescriptions in outpatient settings. While we believe that the factors identified and mechanisms discussed are universal and applicable across different settings, prescriptions in inpatient settings may be influenced by additional factors that are specific to this particular context. This should be investigated in future research. Finally, we did not use ‘antibacterials’ as a keyword in our search. With hindsight, this omission might have inadvertently limited the results of our search, to the extent that relevant articles exist that use this term and might not be discoverable through searching using the terms ‘antimicrobials’ and ‘antibiotics’.

Despite the limitations, we believe that the proposed framework could be useful for underpinning the design of interventions. We offer this analysis as an additional contribution to the research on psychological influences on medical decision-making^15,128,129^ and as a new perspective on the problem of antimicrobial resistance and an opportunity to tackle this problem using a different—behavioural science—approach. In recent years, behavioural science has been increasingly applied in different areas of policy and practice and has shown much potential.^130^ Given that physicians’ decisions to prescribe antimicrobials constitute an important cause of antimicrobial resistance and that, as our analysis shows, these decisions are susceptible to a range of biases known to behavioural scientists, we believe that behavioural science can be an important tool to use when tackling antimicrobial resistance.
